# AlphaFold2 and Deep Learning for Elucidating Enzyme Conformational Flexibility and Its Application for Design

**DOI:** 10.1021/jacsau.3c00188

**Published:** 2023-06-06

**Authors:** Guillem Casadevall, Cristina Duran, Sílvia Osuna

**Affiliations:** † Institut de Química Computacional i Catàlisi (IQCC) and Departament de Química, Universitat de Girona, Maria Aurèlia Capmany 69, 17003 Girona, Spain; ‡ ICREA, Passeig Lluís Companys 23, 08010 Barcelona, Spain

**Keywords:** AlphaFold2, conformational heterogeneity, free energy landscape, enzyme design, deep learning

## Abstract

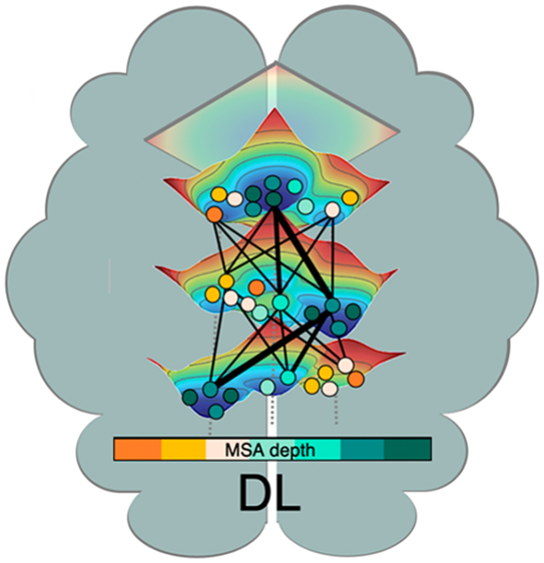

The recent success of AlphaFold2 (AF2) and other deep learning (DL) tools in accurately predicting the folded three-dimensional (3D) structure of proteins and enzymes has revolutionized the structural biology and protein design fields. The 3D structure indeed reveals key information on the arrangement of the catalytic machinery of enzymes and which structural elements gate the active site pocket. However, comprehending enzymatic activity requires a detailed knowledge of the chemical steps involved along the catalytic cycle and the exploration of the multiple thermally accessible conformations that enzymes adopt when in solution. In this Perspective, some of the recent studies showing the potential of AF2 in elucidating the conformational landscape of enzymes are provided. Selected examples of the key developments of AF2-based and DL methods for protein design are discussed, as well as a few enzyme design cases. These studies show the potential of AF2 and DL for allowing the routine computational design of efficient enzymes.

## 1 Introduction

The 60-year problem of knowing the folded structure from the primary sequence of proteins (and enzymes) was thought to be *solved* by the recent success of Alphafold2 (AF2).^1−3^ AF2 is a deep-learning (DL) algorithm that incorporates novel neural network architectures based on the evolutionary, physical, and geometric constraints of protein structures and is able to predict with high levels of accuracy the three-dimensional structure of proteins. AF2 is recognized as one of the milestones in protein structure prediction and has boosted the application of DL methods for many other applications.^4^ Despite the impressive performance of AF2 algorithms in predicting the native lowest in energy structure of enzymes, knowing the single static folded structure is not sufficient for understanding and engineering function, as recently highlighted.^5,6^ As discussed below, another limitation of these methods is that nonprotein parts (i.e., cofactors, substrates, metal ions) are not predicted.

**Figure 1:**
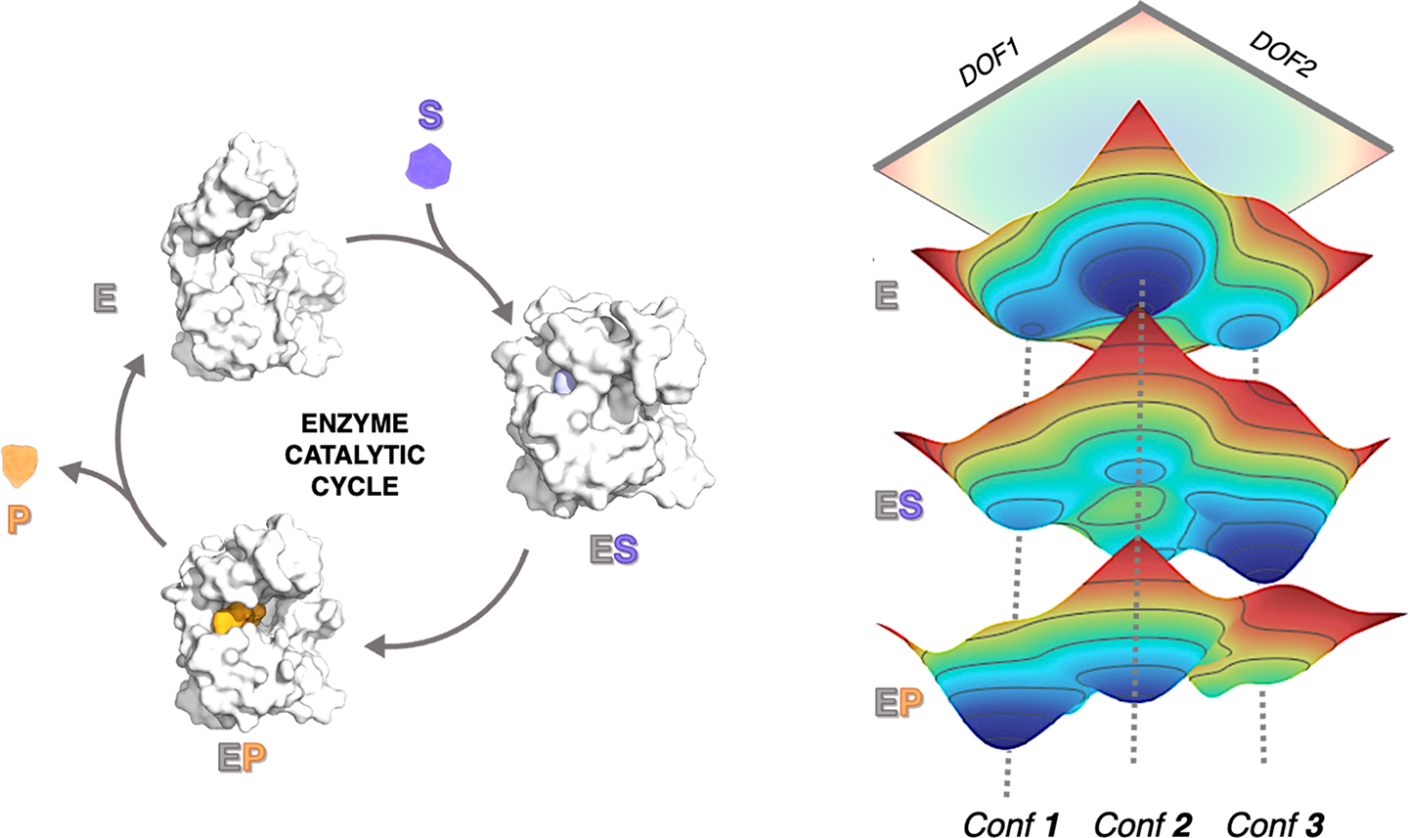
Schematic representation of a catalytic cycle of a model enzyme and associated conformational changes represented in the Free Energy Landscape (FEL) at the different steps: free enzyme (E), enzyme–substrate (ES), and enzyme–product (EP). For FEL reconstruction some key degrees of freedom (DOF) need to be defined, as explained in section 3.

The three-dimensional structure of the enzymes indeed provides very relevant information on the arrangement of the catalytic machinery and structural elements gating the active-site pocket, but understanding enzymatic function requires the exploration of the ensemble of thermally accessible conformations that enzymes adopt in solution. This ensemble of conformations can be represented in the so-called Free Energy Landscape (FEL, see Figure 1 for FELs at different reaction stages),^7^ which displays the relative stabilities of the thermally accessible conformations, as well as the kinetic barriers separating them. Conformational changes that can directly impact catalytic function include side-chain conformational changes in the fast time scale, loop motions often playing a key role in substrate binding/product release in slower time scales, and in some cases allosteric transitions that usually correspond to the slowest processes. The evaluation of the conformational landscapes of natural and evolved enzymes has provided relevant new insights. Experimental X-ray structures and associated B-factors,^8^ room-temperature X-ray experiments,^9,10^ and NMR experiments^11^ have been used to explore the changes in the conformational landscape induced by mutations along several enzyme variants generated with the experimental Directed Evolution technique. From a computational perspective, the reconstruction of the FEL and how this is shifted after mutation provides crucial information for understanding enzyme function (and also for design).^7^

It has been recently shown by different groups that AF2 can be actually tuned to provide multiple conformations of the same protein, which suggests the potential of AF2 for elucidating the conformational landscape of enzymes and proteins.^12,13^ Given the rather low computational cost of AF2, especially if compared to the computationally demanding Molecular Dynamics (MD) simulations, its application for assessing the effect of mutations on the conformational landscape is highly appealing. This could impact the development of AF2-based conformationally focused enzyme designs protocols.^7,14^

Multiple reviews are available in the literature covering the available tools for rationalizing the changes in activity induced by mutations in several enzymes^15−17^ and for rationally designing novel enzymes by means of computational protocols based on Quantum Mechanics (QM), hybrid QM and Molecular Mechanics (QM/MM), Empirical Valence Bond (EVB), MD, and Monte Carlo simulations, or combinations of them.^14^ Instead, in this Perspective we will cover a few of the most recent applications of DL strategies for elucidating enzyme conformational flexibility and for its application for enzyme design.

## 2 The Role of Conformational Dynamics in Enzyme Function and Evolution

Enzymes present highly preorganized active-site pockets with the catalytic residues perfectly arranged for efficiently stabilizing the transition state(s) of a specific reaction.^18,19^ This preorganization is complemented by the enzyme ability to adopt multiple conformations of importance for substrate binding and/or product release. The importance of conformational flexibility was clearly shown with the design of catalytic antibodies presenting an ideal complementary structure to the transition state, which showed a clearly inferior catalytic activity with respect to enzymes.^20^ This shows that efficient catalysis requires not only transition-state stabilization but also the optimization of the conformational ensemble.^14,21^ In fact, the ability of enzymes to adapt and evolve toward novel functions either by natural or laboratory evolution has been connected to their inherent conformationally rich dynamic nature.^7,22−25^ Enzymes display a high degree of flexibility and versatility as shown by their promiscuous side activities^26^ and also their tolerance to evolve toward novel functions.^7,22−25^

Along the enzymatic cycle the following steps take place: (1) first, the substrate(s) bind to the catalytic pocket, which often require and/or induce a change in the conformation of loops and flexible domains regulating the access to the active site;^27,28^ (2) the substrate(s) are activated to facilitate productive formation of the Enzyme–Substrate (ES) complex; (3) this is followed by the stabilization of the transition state(s) for the formation of multiple reaction intermediates and product(s); (4) finally, once the Enzyme–Product (EP) complex is formed the product(s) are released from the pocket, which is often accompanied by conformational changes that initiate the next round of the catalytic cycle. All of these steps are essential for maximizing catalytic activity by optimized throughput of the overall pathway. The binding of the substrate for ES formation can also modulate the conformational landscape as shown for the multienzyme complex pyruvate dehydrogenase complex.^29^ In Figure 1, the conformational changes that take place along the catalytic itinerary of the enzyme adenylate kinase (AdK) is shown as a model. The catalytic cycle involves the conformational change from open to closed structures of a lid that covers the active site. The computational evaluation of the chemical steps along the catalytic itinerary (steps 2 and 3) require the use of QM, hybrid QM/MM, and EVB, which are too expensive to be applied for analyzing the conformational changes taking place through the cycle and the processes of substrate binding and product release (steps 1 and 4).^7,14,15^ This explains the large available number of computational approaches developed along the years. Current computational strategies put mostly the focus to only some of the above-mentioned features, in part explaining the often low success in achieving high levels of enzymatic activity.^14^

## 3 Computational Reconstruction of the Free Energy Landscape

The ensemble of conformations that enzymes adopt in solution can be represented in the free energy landscape (FEL). The free energy (*G*) is proportional to the negative logarithm of the population distribution in *k*_B_*T* units; thus, a maximum in this distribution is a minimum in the FEL. The FEL therefore provides crucial information on the thermodynamics (i.e., which are the lowest in energy conformations at a given set of conditions) and the kinetics for the conformational transitions. These energy barriers separating the different minima will determine the time scale of the conformational exchange: fast conformational changes occur in the picosecond to microsecond time scales (this is the case of loop motions crucial for enzyme catalysis), whereas slow motions will take place in millisecond to seconds.

**Figure 2:**
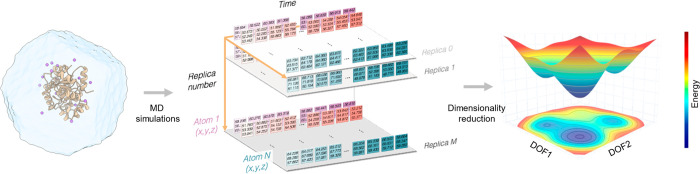
Schematic representation of the Free Energy Landscape (FEL) reconstruction process. The high dimensional data of the MD simulations needs to be reduced and projected into a set of key collective degrees of freedom (DOF) for probability distribution calculation to reconstruct the FFEL.

Enzymes can be captured in different conformational states by means of X-ray, room-temperature, and time-resolved X-ray, cryo-EM, NMR, and biophysical techniques can be applied for providing complementary kinetic information.^30^ These multiple conformations of the same enzyme deposited in the protein data bank (PDB) played an important role in AF2 training but also for the AF2 application for assessing the conformational heterogeneity of biological systems (as discussed below). Computational methods are particularly appropriate for reconstructing the FEL: MD simulations sample the population distribution by integrating Newton’s laws of motion. By defining a reduced set of collective degrees of freedom (DOFs) the high dimensional data obtained in the MD runs can be projected for probability distribution calculation and thus FEL reconstruction (see Figure 2). The selection of the reduced set of DOFs can be made manually or automatically by means of different dimensionality reduction schemes.^7,31^ The accurate exploration of the conformational changes for FEL reconstruction requires extensive MD simulations, and depending on the time scale of the conformational transitions enhanced sampling techniques need to be applied.^7,14,15^ These techniques have a high computational cost associated with them (from weeks to many months of simulations), which limits the applicability of these strategies for computationally designing and ranking enzyme designs.

## 4 Application of AF2 for Capturing Conformational Heterogeneity

**Figure 3:**
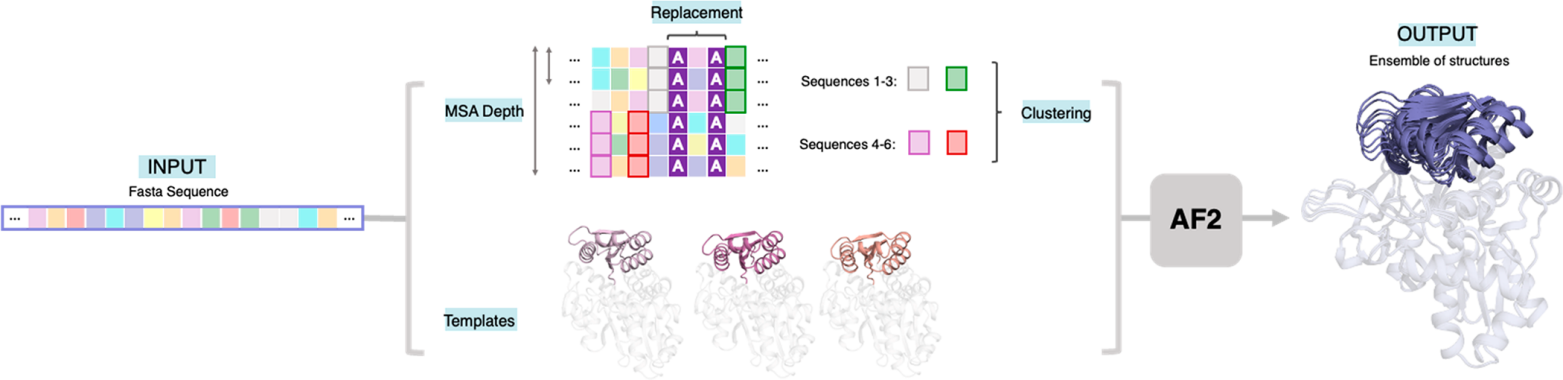
Overview of strategies developed for predicting alternate states with AlphaFold2 (AF2). As done in del Alamo et al.^12^ the Multiple Sequence Alignment (MSA) depth can be altered, some of the MSA positions can be masked as shown by Stein and McHaourab,^13^ and the MSA can be clustered as in the Kern and Ovchinnikov preprint paper.^37^ The provided set of templates can also be changed as done in some cases in del Alamo et al.^12^ and also in our recent publication.^34^

The standard AF2 protocol requires the primary sequence of the enzyme, a multiple sequence alignment (MSA) generated with information on evolutionary related proteins, and the 3D coordinates of a small number of homologous structures named templates (see Figure 3). Although AF2 was designed to predict single *static* structures, some recent papers have shown that by reducing the depth of the input MSAs used in the AF2 algorithm (in addition to decrease the number of recycles) accurate models in multiple conformations can be generated.^12,13,32^ In particular, del Alamo and coworkers showed that multiple conformations of transporters and G-protein-coupled receptors can be obtained by altering the AF2 pipeline and providing a reduced number of MSA sequences (as low as 16 sequences only).^12^ They generated up to 50 different models of each protein receptor for each MSA size, as opposed to the standard AF2 protocol that provides conformationally homogeneous and nearly identical models. Interestingly, they observed limited conformational sampling for proteins that were contained in the AF2 training set. In another study, Stein and McHaourab reported a universal method for biasing the models generated by AF2 based on the replacement of specific residues within the MSA to alanine or another residue.^13^ AF2 was used to generate initial models, and the MSA was modified based on possible contact points in the initial structures, prior structural information, or regions of uncertainty within the main structure. They found that the replacement of certain amino acid columns to alanine or other residues turns the attention of the network to other parts of the MSA allowing for AF2 to find alternative conformations based on other coevolved residues. One of the provided examples is AdK that undergoes a large-scale conformational change of a lid and a flap that gate the active site, as revealed by the unbound and inhibitor-bound crystal structures (see Figure 1). By masking some residues and replacing them to alanine, closed and open strcutures of AdK were obtained. Although none of the AF2 open structures reached the level of opening of the crystal structure (PDB: 4AKE), the set of generated AF2 models displaying a different level of closure showed the potential of the methodology for predicting alternate conformations describing the conformational heterogeneity of the systems.

**Figure 4:**
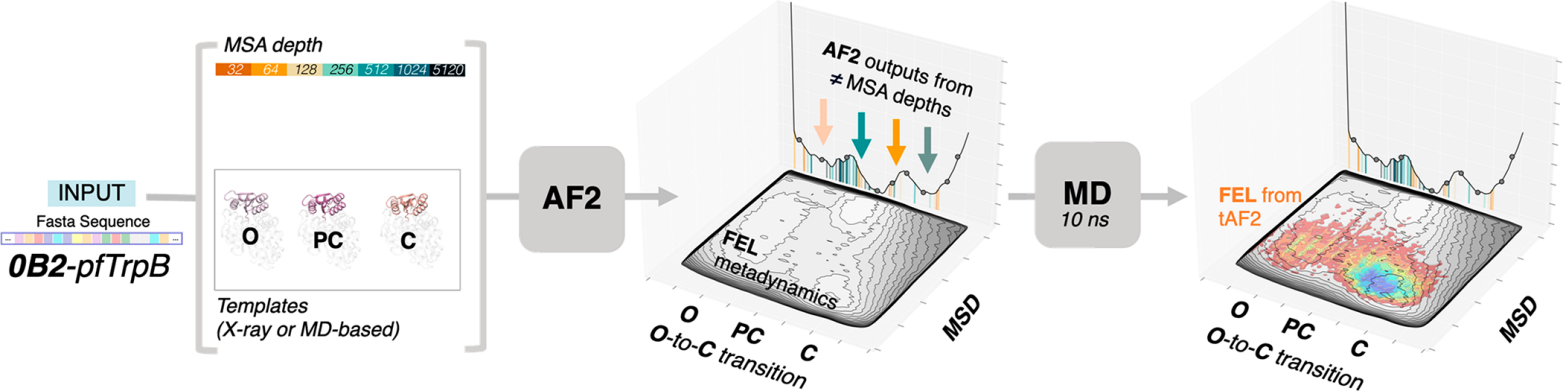
Our template-based AF2 (tAF2) approach for estimating the conformational heterogeneity. Different Multiple Sequence Alignment (MSA) depths and set of templates taken from either selected X-ray structures or Molecular Dynamics (MD) snapshots are provided to AF2.^34^ The multiple output models generated by AF2 at the different MSA depths shown as vertical lines in the central plot are then subjected to short MD simulations for FEL reconstruction. The new FEL generated from the 10 ns MD simulations starting from the ca. 1000 AF2 outputs at different MSA is shown in a blue to red colormap on top of the computationally reconstructed FEL obtained via well-tempered multiple-walker metadynamics simulations (in gray).^33,34^ The x and *y* axis of the reconstructed FELs indicate the Open-to-Closed (O-to-C) transition of the COMM domain of TrpB that covers the active site, and the Mean Square Deviation (MSD) from the path of the generated O-to-C structures, respectively.^33−35^ The input sequence is the 0B2-*pf*TrpB variant.^38^ Reproduced with permission from ref (34). Copyright 2022 John Wiley & Sons, Inc.

Inspired by these previous publications showing AF2’s ability to sample additional conformational states, we developed a template-based AF2 approach to assess the conformational heterogeneity and how this is altered by mutations on the β subunit of several tryptophan synthase enzymes (TrpB, see Figure 4).^33−35^ As done in the work of del Alamo et al.,^12^ we tested the effect of reducing the provided number of sequences in the MSA, but we additionally assessed how AF2 predictions are altered when different templates displaying multiple conformational states are provided.^34^ We tested the template-based AF2 pipeline by providing either X-ray based or conformations extracted from MD simulations as templates. With these settings AF2 revealed major differences in the conformational landscapes among the analyzed systems. Interestingly, this was further demonstrated by running multiple short MD simulations from the set of AF2 structures and reconstructing the associated FELs (Figure 4). The comparison of the generated FEL from the template-based AF2 predictions were in line with the computationally expensive FELs generated with well-tempered multiple-walker metadynamics simulations. This is exciting as it shows the potential of AF2 for rapidly and accurately assessing the FELs of different systems, which could be applied for conformationally driven enzyme design approaches.^7,14^ The multiple outputs obtained via AF2 at different MSA depths were also recently combined with Reweighted Autoencoded Variational Bayes for Enhanced (RAVE) sampling.^36^

In a recent preprint paper, Kern and Ovchinnikov showed that clustering the input MSA by sequence similarity allows AF2 to visit multiple conformational states of some metamorphic proteins known to display large conformational changes.^37^ They also identified two mutations that according to AF2 predictions could switch the circadian rhythm protein KaiB between the two major conformational states. Their developed methodology was also applied for searching for alternative conformational states in other proten families and found a putative alternate state for the oxidoreductase DsbE. These computational predictions were, however, not tested experimentally in the published preprint paper.

## 5 Application of AF2 and Other Deep Learning Techniques for Protein and Enzyme Design

Inspired by the AF2 approach, other DL techniques have been recently developed for elucidating the folded structure of enzymes and providing some metrics to be potentially used for protein design. The field is advancing fast, and the number of DL strategies developed especially for protein design is constantly increasing. In this section, we aim to provide a brief overview of the most representative techniques developed, and we put special emphasis to those strategies that are particularly relevant for enzyme design. Some recent reviews focused on structure-based protein design with DL strategies are available,^39,40^ as well as a review related to the design of more stable enzymes.^41^

These available strategies for structure prediction can be classified depending on the number of input parameters used: those that require the input query sequence, MSA, and set of templates for accurate predictions and those that predict the folded structure based on the input sequence only. Similarly to AF2, the RoseTTAFold (RF) algorithm developed almost at the same time as AF2 requires an MSA and a set of initial templates to make accurate predictions of the folded structure. RF showed improved accuracy toward protein–protein complex prediction as compared to AF2 and AF2 multimer.^42,43^ OpenFold2 was also developed to replicate the AF2 algorithm and make it accessible to the structural biology community.^44^ AlphaLink was also introduced to incorporate experimental distance restraint information, thus generating a modified version of the AF2 network architecture.^45^

Sequences contain implicit information about the enzyme structure and function, as the position of each amino acid in the sequence is determined by the spatial arrangement and the possible interactions established between them. The main advantage is the comparison of sequences is computationally cheap (at least as compared to physics-based approaches) and provides crucial information about the most frequent residues at each position, conservation score, and correlated mutation pairs that have emerged during evolution. Covarying mutations have been associated with function, tertiary contacts, and binding. It has also been shown that the use of language models previously used for Natural Language Processing (NLP) could be applied in the context of the biology language to generate “content-aware” data representations from large-scale sequence data sets. This is the case of ESM-2, which corresponds to the largest language model of protein sequences developed to date.^46^ ESMFold was then developed, which was found to perform end-to-end folded structure predictions with similar accuracies to AF2, *albeit* at an order of magnitude faster.^46^ OmegaFold (OF) is another end-to-end structure prediction algorithm developed that combines a pretrained language model and a geometrical transformer model for reconstructing the structure.^47^ Similarly to ESMfold, OF only needs the input sequence and is 10-fold faster than AF2 and RF. More importantly, OF was found to do a better job in predicting the folded structure of orphan proteins, i.e., those proteins that do not have any assigned functional family.

Apart from the different methodologies developed to predict the folded structure of proteins, different NLP and deep-learning architectures have been developed to generate new non-natural sequences. These different strategies have targeted different objectives that range from generating new sequences for maintaining some natural activities^48,49^ to imagining new folds and sophisticated symmetric assemblies,^50^ among others.

The generative language models ProGen and ProGen2 trained on millions of raw protein sequences were developed to generate de novo artificial proteins that express well and maintain enzymatic function.^51,52^ ProtGPT2 is an unsupervised language model that can generate new sequences based on the principles of natural ones.^53^ Similarly, variational autoencoders trained on a data set of luciferase-like oxidoreductases were also used to generate new sequences maintaining the luciferase activity.^49^ ProteinGAN, which is based on a self-attention-based variant of the generative adversarial network, learns natural protein sequences for generating new functional variants.^54^ The conditional language model ZymCTRL trained on the BRENDA database of enzymes has also been recently developed, which is able to provide new artificial enzymes within a user-defined Enzyme Classification (EC)-based enzymatic class.^55^ Language models have also been used to obtain a set of sequences that are likely to fold into a given desired structure. This is, for instance, the case for recently developed LM-Design^56^ and ProteinDT^57^. Yu and co-workers have recently developed CLEAN based on contrastive learning that is able to assign EC numbers to a given sequence.^58^

The transform-restrained Rosetta (trRosetta) was developed by the Baker lab in 2020 to design a variety of proteins by randomly modifying the starting sequences to find sharply predicted residue–residue interdistance maps.^59^ The combination of trRosetta and the physics-based Rosetta was shown to provide more funneled energy landscapes: trRosetta was used to disfavor alternative states, and high-resolution Rosetta was used for creating a deep energy minimum at the designed target structure.^60^ Small β barrel proteins and proteins with discontinuous functional sites were developed with trRosetta.^61,62^ Recently, Dauparas and co-workers developed a method called ProteinMPNN, which is a graph neural network that was found to rescue previously failed designs targeted with Rosetta or AF2.^63^ ProteinMPNN was recently applied to generate de novo luciferases.^64^ MutComput is a convolutional neural network (CNN) that was successfully applied for designing new hydrolases for poly(ethylene terephthalate) depolymerization.^65^ Another more recent CNN for protein sequence design was provided by Anand et al. to generate a de novo TIM-barrel protein backbone.^66^ Holographic CNNs have also been developed to learn the shape of protein microenvironments to predict the impact of mutations on stability and binding of protein complexes.^67^

Different protocols based on the use of AF2 for predicting the structure of the generated sequences and use the output AF2 metrics for the design of new proteins have also been developed. The AlphaDesign computational framework was constructed to enable the rapid prediction of completely novel protein monomers starting from random sequences.^68^ The potential application of AlphaDesign for designing proteins that bind to prespecific target proteins was also shown. AF2 was also used for the rapid and accurate fixed backbone design of sequences that are strongly predicted to fold to a specific backbone.^69^ The Baker lab combined ProteinMPNN with AF2 to design closed repeat proteins with central pockets^50^ and generate symmetric protein assemblies.^50^ Similarly, RF instead of AF2 was used for designing high-affinity protein binders^70^ or proteins with prespecified functional motifs.^71^ RF has also the potential to predict the effect of mutations on protein function.^72^

The RF-based diffusion model (named RF*diffusion*) has been recently developed by the Baker lab.^73^ RF*diffusion* can very rapidly and accurately design topology-constrained protein monomers, protein binders, symmetric oligomers, metal-binding proteins, and even enzyme scaffolds containing specific active-site residues.^73^ The performance of RF*diffusion* outperforms hallucination in terms of success rate, accuracy, and speed. Even though RF*diffusion* does not explicitly consider the substrate molecule, it can be implicitly modeled using an external potential to guide the generation of the active-site pocket.

As mentioned in the Introduction, catalytic function requires substrate binding and product release, and in many cases enzymatic activity is dependent on cofactor and metal ion binding. In this direction, different strategies based on DL have also been generated to dock ligands, substrates, and missing cofactors into potential pockets. AlphaFill uses sequence and structural similarity to include the missing organic molecules and metal ions into the AF2 models.^74^ The diffusion generative model DiffDock was designed to dock small molecules into potential protein pockets. This strategy was shown to outperform previous traditional and DL docking protocols.^75^ Meller and co-workers also developed an AF2-based strategy to find cryptic pockets.^76^ DL has also been applied for finding potential location sites of transition metals in proteins (Metal1D and Metal3D).^77^ The coevolution based MetalNet pipeline has also been recently created to predict potential metal-binding sites.^78^

## 6 Outlook and Future Prespectives

Enzyme catalysis is a complex multidimensional process that requires the optimal sequence and structure for allowing substrate(s) binding, catalyzing the chemical steps and product(s) release, and optimizing the multiple conformations needed for developing its function. This high complexity makes the task of enzyme design, especially toward non-natural reactions or substrates in high efficiencies very challenging. The selected examples highlighted in this review show the potential of DL techniques to generate new functional variants mostly within the allowed biological constraints of the sequence space. The application of DL strategies for computational enzyme design for any target reaction and non-natural substrate is only at its beginning. For many years, the lack of precision in incorporating the desired active-site residues into protein scaffolds in computational enzyme design has been considered one of the many limitations of the overall process. This point, however, seems to be solved with the recent RosettaFold-based diffusion model developed by the Baker lab. The incorporation of the QM-based models of the enzyme active site into new non-natural scaffolds specifically designed to hold the functional motifs in place might no longer be the limiting factor, but instead predicting which scaffolds might be more appropriate for the optimization of the conformational ensemble for efficient catalysis will most likely be essential. Considering the huge advances especially in the field of structure prediction and protein design seen these recent years, the combination of DL methods with physics-based approaches will play a key role the coming years for finding optimal solutions for the rational and routine design of highly efficient and stable enzymes for non-natural reactions and substrates.

## Abbreviations

AF2: AlphaFold2
DL: Deep Learning
FEL: Free Energy Landscape
MD: Molecular Dynamics
